# Promoter Usage and Dynamics in Vascular Smooth Muscle Cells Exposed to Fibroblast Growth Factor-2 or Interleukin-1β

**DOI:** 10.1038/s41598-018-30702-4

**Published:** 2018-09-03

**Authors:** Ahmad M. N. Alhendi, Margaret Patrikakis, Carsten O. Daub, Hideya Kawaji, Masayoshi Itoh, Michiel de Hoon, Piero Carninci, Yoshihide Hayashizaki, Erik Arner, Levon M. Khachigian

**Affiliations:** 1 Vascular Biology and Translational Research, School of Medical Sciences, University of New South Wales, Sydney, 2052 Australia; 2 RIKEN Center for Life Science Technologies (Division of Genomic Technologies) (CLST DGT), 1-7-22 Suehiro-cho, Tsurumi-ku, Yokohama, Kanagawa 230-0045 Japan; 3 Department of Biosciences and Nutrition and Science for Life Laboratory, Karolinska Institutet, SE-141 86 Stockholm, Sweden; 4 RIKEN Center for Integrative Medical Sciences, Yokohama, Kanagawa 230-0045 Japan; 5 RIKEN Omics Science Center (OSC), 1-7-22 Suehiro-cho, Tsurumi-ku Yokohama, 230-0045 Japan; 6 RIKEN Preventive Medicine and Diagnosis Innovation Program (PMI), 2-1 Hirosawa, Wako-shi, Saitama 351-0198 Japan; 7 Preventive Medicine and Applied Genomics Unit, RIKEN Center for Integrative Medical Sciences, Yokohama, Kanagawa 230-0045 Japan; 8 Laboratory for Applied Computational Genomics, RIKEN Center for Integrative Medical Sciences, Yokohama, Kanagawa 230-0045 Japan; 9 Laboratory for Transcriptome Technology, RIKEN Center for Integrative Medical Sciences, Yokohama, Kanagawa 230-0045 Japan; 10 Laboratory for Applied Regulatory Genomics Network Analysis, RIKEN Center for Integrative Medical Sciences, Yokohama, Kanagawa 230-0045 Japan

## Abstract

Smooth muscle cells (SMC) in blood vessels are normally growth quiescent and transcriptionally inactive. Our objective was to understand promoter usage and dynamics in SMC acutely exposed to a prototypic growth factor or pro-inflammatory cytokine. Using cap analysis gene expression (FANTOM5 project) we report differences in promoter dynamics for immediate-early genes (IEG) and other genes when SMC are exposed to fibroblast growth factor-2 or interleukin-1β. Of the 1871 promoters responding to FGF2 or IL-1β considerably more responded to FGF2 (68.4%) than IL-1β (18.5%) and 13.2% responded to both. Expression clustering reveals sets of genes induced, repressed or unchanged. Among IEG responding rapidly to FGF2 or IL-1β were FOS, FOSB and EGR-1, which mediates human SMC migration. Motif activity response analysis (MARA) indicates most transcription factor binding motifs in response to FGF2 were associated with a sharp induction at 1 h, whereas in response to IL-1β, most motifs were associated with a biphasic change peaking generally later. MARA revealed motifs for FOS_FOS{B,L1}_JUN{B,D} and EGR-1..3 in the cluster peaking 1 h after FGF2 exposure whereas these motifs were in clusters peaking 1 h or later in response to IL-1β. Our findings interrogating CAGE data demonstrate important differences in promoter usage and dynamics in SMC exposed to FGF2 or IL-1β.

## Introduction

Vascular smooth muscle cells (SMC) reside in normal arteries in a growth quiescent contractile state and respond to growth factors such as fibroblast growth factor-2 (FGF2) or pro-inflammatory cytokines such as interleukin-1β (IL-1β) which can arise from cellular and matrix trauma, infection, inflammation or platelet activation^1^. FGF2 and IL-1β have long been recognised as key mediators in SMC pathobiology. For example, neutralizing FGF2 antibodies reduce SMC proliferation induced by balloon catheterization by approximately 80%^2^. FGF2-driven SMC growth after balloon injury of carotid arteries is dependent on endogenous heparan sulfate proteoglycans^3^. Moreover the sulphated oligosaccharide PI-88 which binds FGF2 and blocks SMC proliferation inhibits intimal thickening after balloon injury^4^. Inhibition of FGF receptor signalling with the tyrosine kinase inhibitor SU5402 attenuates SMC and macrophage accumulation in atherosclerotic lesions in ApoE-deficient mice^5^. IL-1β can have autocrine growth effects on SMC^2,6^. The lack of IL-1β reduces the severity of atherosclerosis in ApoE-deficient mice^7^. IL-1β is produced by endothelial cells and macrophages in coronary arteries from patients with ischemic heart disease^8^. Recent studies have shown that numerous NLRP3 inflammasome components including IL-1β are markedly expressed in human atherosclerotic plaques and that IL-1β is released in freshly isolated human carotid plaques by lipopolysaccharide and cholesterol crystals^9^. Hence FGF2 and IL-1β are model agonists of growth factor and cytokine mediated SMC growth.

SMC dedifferentiate toward a proliferative and migratory state and can themselves produce growth factors, cytokines and matrix components^10^. These cellular changes involve the coordinated expression of a wide range of genes, including immediate-early genes (IEG) within minutes of cellular stimulation and typically do not require *de novo* protein synthesis for gene expression^11^. The Functional ANnoTation Of the Mammalian genome (FANTOM) consortium has redefined our understanding of dynamic changes in gene expression across a broad range of cell types. FANTOM5 recently investigated *en masse* the dynamic regulation of promoters and enhancers in 19 human and 14 mouse time courses and proposed a generalizable model whereby enhancer transcription is the earliest event in cells undergoing transcriptional change during differentiation or activation^12^. This was facilitated by single molecule Cap Analysis of Gene Expression (CAGE) analyses where reverse-transcribed first strand cDNAs corresponding to capped 5′-end of RNAs were captured and sequenced by a single molecule sequencer without any steps of PCR amplification^13^. The produced sequences are then mapped to the genome and allow identification of transcriptional start sites (TSS) of capped RNAs (mRNAs and lncRNAs). Generating CAGE libraries at multiple time points after stimulation has allowed us to study dynamic expression^14^.

While IEG have long been recognised as early regulators of cellular growth and differentiation, with many serving as transcriptional regulators, receptor components and cytoskeletal and secreted proteins, until FANTOM5, our understanding of dynamic changes and generality of expression was largely incomplete. FANTOM5 redefined our understanding of promoter activation by defining 8 distinct subtypes of dynamic expression. Here we describe the dynamic expression of specific genes in SMC responding to a prototypic growth factor or pro-inflammatory cytokine and focus on transcriptional rather than cellular changes. CAGE data sourced from FANTOM5 has enabled appreciation of differences in promoter usage and dynamics to FGF2 or IL-1β.

## Results

### Comparison of dynamically expressed genes upon FGF2 and IL-1β treatment

**Figure 1:**
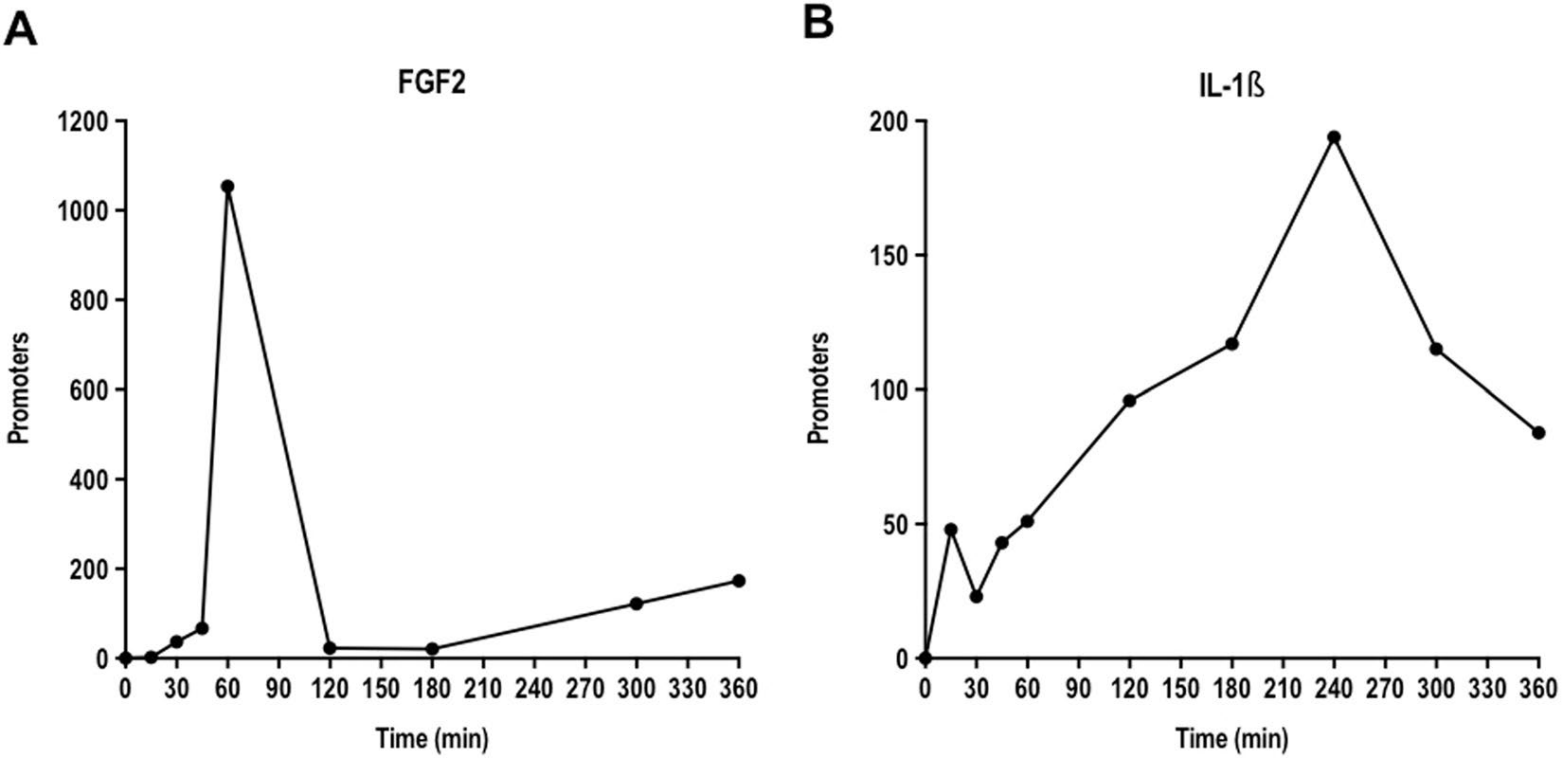
Numbers of promoter demonstrating pairwise significant change at each time point in response to FGF2 or IL-1β relative to unstimulated cells. Promoter responses to (**A**) FGF2, or (**B**) IL-1β with significant pairwise change (5% FDR threshold by edgeR software) at 15, 30, 45, 60, 120, 180, 240 (IL-1β only), 300 or 360 min relative to unstimulated cells.

Of the 1871 promoters demonstrating a significant response (i.e. differentially expressed in at least one of all the pairwise comparisons of all time points up to and including 6 h) to FGF2 or IL-1β in the SMC samples as described in ref.^15^ considerably more promoters responded to FGF2 (68.4%) as compared to IL-1β (18.5%) and 13.2% responded to both FGF2 and IL-1β (Supplementary Table 1). We plotted the number of promoters demonstrating pairwise significant change (i.e. promoters that are significantly differentially expressed at 5% false discovery rate (FDR) threshold) at each time point in response to FGF2 or IL-1β relative to unstimulated cells. There was a sharp inducible response at 1 h with FGF2 (Fig. 1A) whereas with IL-1β there was generally an inducible sustained later response peaking at 4 h (Fig. 1B) noting that response in this regard does not refer to magnitude of change but rather that there is significant pairwise change relative to unstimulated cells or within the 6 h time course (Supplementary Table 1).

### Clusters of expression in response to FGF2 and IL-1β by k-means analysis

**Figure 2:**
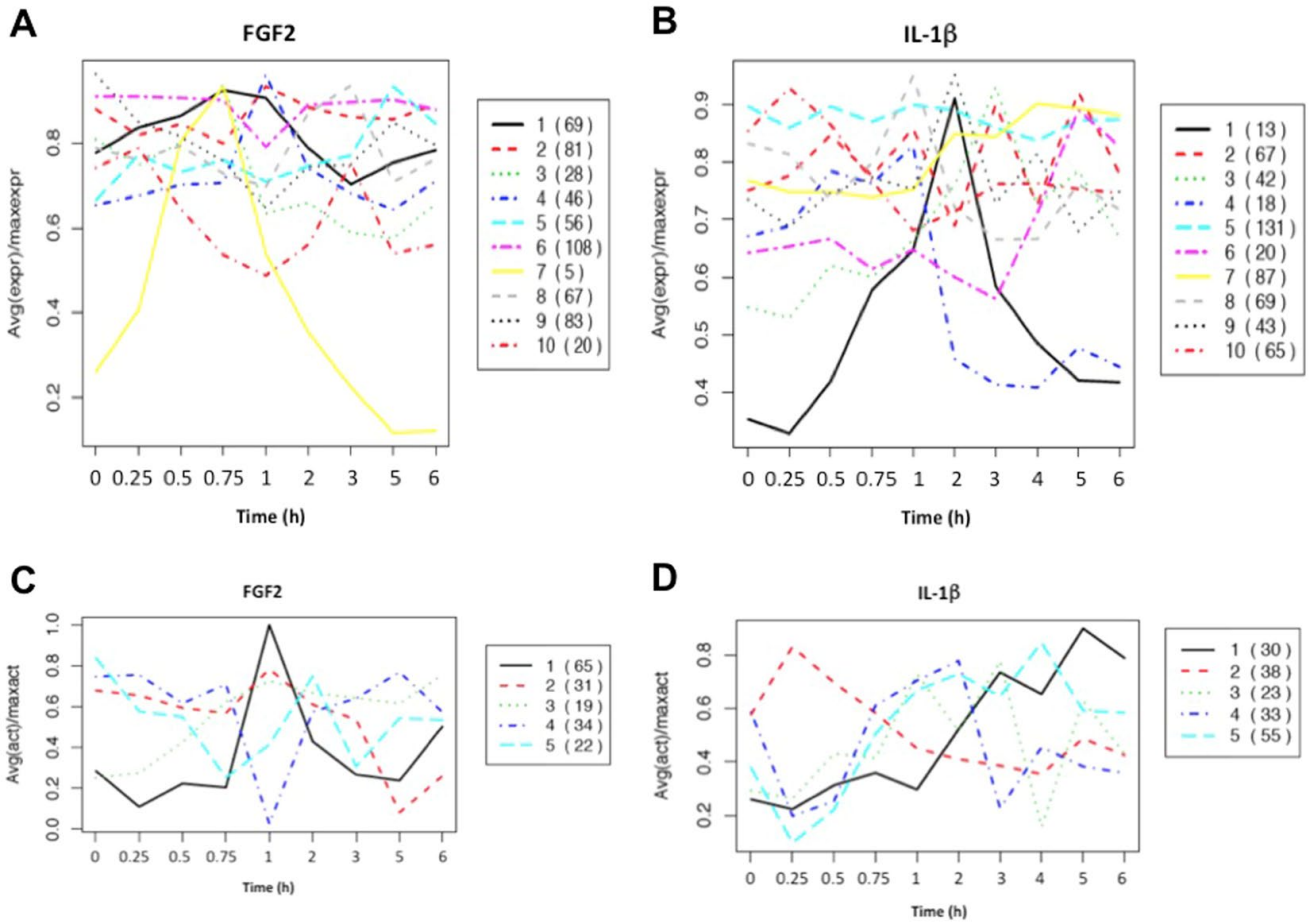
Clusters of altered transcription factor expression and motif activities in SMC exposed to FGF2 or IL-1β. Clusters of altered TF expression in response to (**A**) FGF2 or (**B**) IL-1β. Clustering of motif activities in response to (**C**) FGF2 or (**D**) IL-1β. Numbers in parentheses denote the number of TFs or motifs within the cluster.

Arner *et al*. identified differences in promoter usage and promoter dynamics in SMC exposed to FGF2 and IL-1β by analysing CAGE data sourced from these cells having regard to the dynamic profile classifications set out in detail in ref.^12^. To obtain a more generalizable view of these changes in promoter activity and group sets of changes into clusters, we performed k-means cluster analysis. This was performed for both FGF2 and IL-1β treated samples and we focused the analysis on promoters belonging to TFs^15^ to identify patterns. The mean profiles of the resultant clusters were plotted as a time series. A range of profiles were obtained showing waves of coordinated expression with time. For example, in SMC exposed to FGF2, expression in Clusters 3, 4 and 7 peaks at 45–120 min, yet expression in Cluster 10 dips within 1 h before increasing and peaking at 3 h (Fig. 2A) and Cluster 6 is relatively unchanged but for a slight dip in expression at 1 h (Fig. 2A). FOS, FOSB and EGR-1 are prototypic IEG encoded TFs. These genes appear together with ATF3 and CSRNP1 in Cluster 7 that peaks at 45 min in the FGF2 response (Fig. 2A). In SMC exposed IL-1β, Clusters 1, 4, 8 and 9 peak at 45–120 min (Fig. 2B). EGR-1 and FOS appear in Cluster 4 and FOSB in Cluster 1 (Fig. 2B). In contrast, Cluster 3 (comprising genes such as NFKB1 and NCOA7) increases steadily peaking at 3 h and Cluster 5 is unchanged (Fig. 2B). Specific genes in the various clusters are listed in Supplementary Table 2.

K-means clustering also identified several other genes implicated in SMC growth and differentiation. Among these, Kruppel-like zinc-finger TFs were particularly represented. KLF2, which is required for SMC migration^16^ is found in Cluster 3 of FGF2-treated SMC (Fig. 2A) and Cluster 4 of IL-1β-treated SMC (Fig. 2B). KLF3, which physically associate and synergies with serum response factor (SRF) and transcriptionally regulates muscle gene^17^ is found in Cluster 4 of FGF2-treated SMC (Fig. 2A) and Cluster 1 of IL-1β-treated SMC (Fig. 2B). KLF5 (also known as BTEB2 and IKLF), which is induced in activated SMC and implicated in the time to restenosis^18^, is found in Cluster 8 of FGF2-treated SMC (Fig. 2A) and Cluster 3 of IL-1β-treated SMC (Fig. 2B). YY1, which we showed inhibits SMC proliferation^19^ is found in Cluster 6 of FGF2-treated SMC (Fig. 2A) and Cluster 5 of IL-1β-treated SMC (Fig. 2B). k-means cluster analysis highlights the transient regulation of gene expression over time in response to FGF2 and IL-1β, and demonstrates that in response to agonist exposure, there were clusters of genes induced, repressed or unchanged.

### Egr-1 mediates human SMC migration

**Figure 3:**
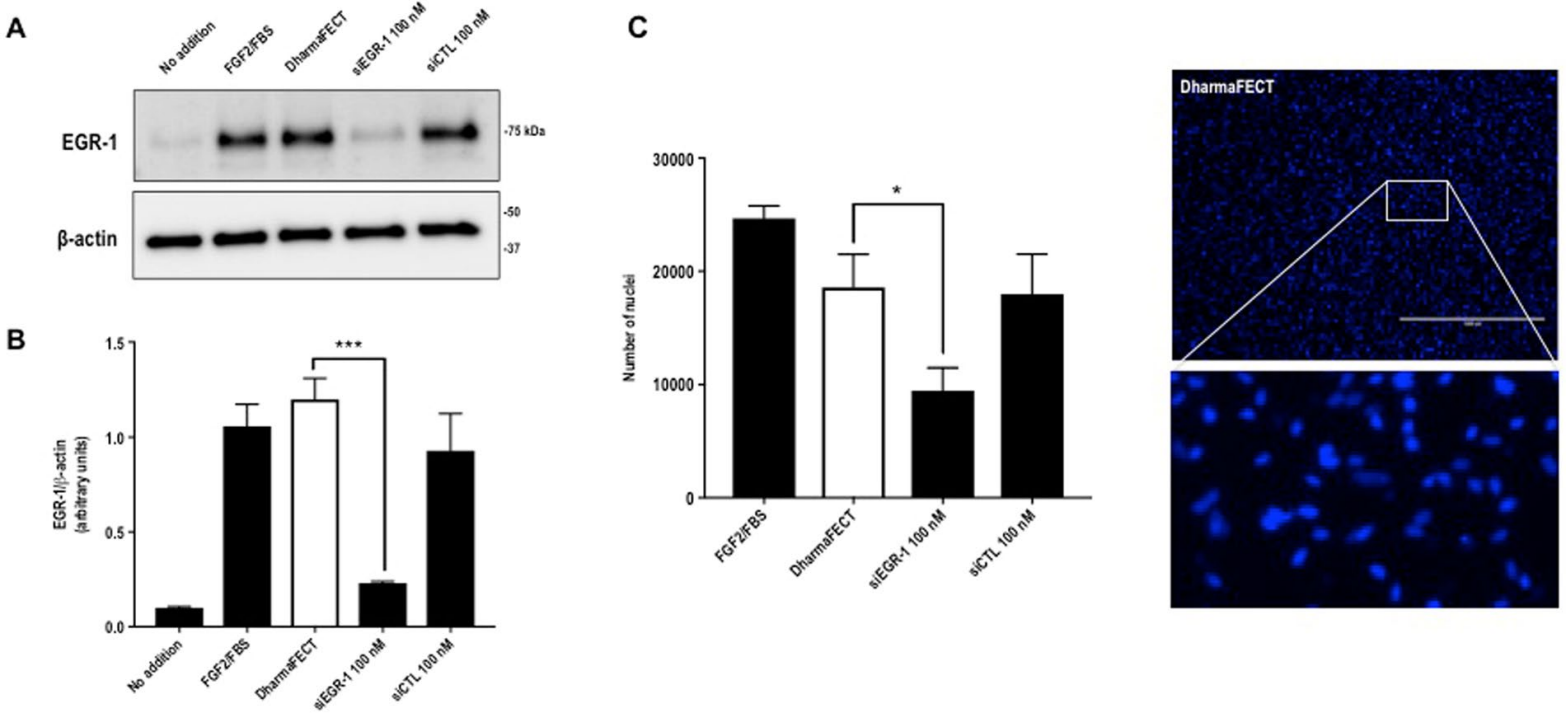
EGR-1 mediates migration in SMC exposed to FGF2. (**A**) SMC rendered growth quiescent in Waymouth’s medium containing 0.05% FBS were treated with 100 nm siRNA, siCTL or DharmaFECT then incubated with 50 ng/ml FGF2 for 1 h. Western bloting was performed with total cell lysates. Each blot is representative of 2 independent experiments. (**B**) Band intensity from 3 independent experiments was quantified using NIH Image J and the EGR-1 intensity expressed as a proportion of β-actin intensity per experiment. Two of the three blots were imaged under identical parameters on a LAS 4000 imager. Error bars represent SEM. Statistical significance was assessed by one-way ANOVA. (**C**) SMC in Waymouth’s containing 20% FBS were seeded into 24-well plates fitted with 0.8 µm Transwell inserts. After 48 h, the medium was changed to Waymouth’s containing 5% FBS and the cells were incubated for 48 h. siRNA, siCTL or DharmaFECT alone was added to the upper chamber at 100 nM and ratio of 1:2 in Waymouth’s medium containing 5% FBS without antibiotics. The medium in the lower chamber was changed to Waymouth’s containing 50 ng/ml FGF2 in 5% FBS. The cells were left for 48 h. Nuclei were quantified using NIH Image J software. Data represents the mean ± SEM of the means of 4 independent experiments. Statistical significance was assessed by one-way ANOVA. *P < 0.05, ***P = 0.0001. A representative image of DAPI stained nuclei from the DharmaFECT group and an enlargement are shown. The scale bar represents 1000 µm.

While Egr-1 plays a positive regulatory role in vascular pathobiology^20^, its role mediating human SMC responsiveness to FGF2 is less well understood. CAGE analysis indicates that Egr-1 levels are induced by FGF2 peaking within 30–60 min and return to basal levels by 6 h (Supplementary Fig. 1A,B). Small interfering RNA (siRNA) targeting Egr-1 inhibited Egr-1 expression upon FGF2 exposure of human SMC within 1 h (Fig. 3A,B). In contrast, control siRNA or Dharmafect carrier had no such inhibitory effect (Fig. 3A,B). SMC migration was reduced by approximately 50% following transfection with Egr-1 siRNA whereas cells transfected with control siRNA or that were simply exposed to the carrier alone had no such effect (Fig. 3C). These data demonstrate the regulatory role of Egr-1 in human SMC migration. This follows our earlier demonstration that Egr-1 controls the reparative response of human SMC to mechanical injury^21^.

### Motif activity response analysis (MARA) of the response to FGF2 and IL-1β

The coordinated gene regulatory response to a given agonist can also be globally analysed using MARA which identifies genes that share putative nucleotide recognition elements (or motifs) in their promoters. Using this technique^22^, we can infer that sets of genes with shared recognition motifs are coregulated by TFs recognising that motif. MARA was used to link TF binding motifs with the dynamic response to FGF2 or IL-1β. Cluster analysis revealed that most motifs in SMC exposed to FGF2 were associated with a sharp inducible response at 1 h (Fig. 2C, Cluster 1), whereas in SMC exposed to IL-1β, most motifs were associated with a biphasic response initially dipping at 15 min and peaking generally later than the FGF2 response between 1–5 h (Fig. 2D, Cluster 5) even though motifs in Clusters 1, 3, 4 also demonstrated peaks between 1–3 h (Fig. 2D). A list of individual motifs included in each of the 5 clusters in response to FGF2 and IL-1β is provided in Supplementary Table 3A,B, respectively. Cluster 1 in the FGF2 response contains motifs for FOS_FOS{B,L1}_JUN{B,D} and EGR-1..3 (Supplementary Table 3A), while Cluster 4 in the IL-1β response contains motifs for EGR-1..3 and Cluster 5 for FOS_FOS{B,L1}_JUN{B,D} (Supplementary Table 3B). Determination by MARA of a sharp inducible response at 1 h is supported by the fact that there are many IEG and other genes sharply induced at 1 h by the different agonists. FOS^23,24^, FosB^24^ and EGR-1^25,26^ are strongly induced by vascular injury in rats within 1 h and the induction is transient.

**Figure 4:**
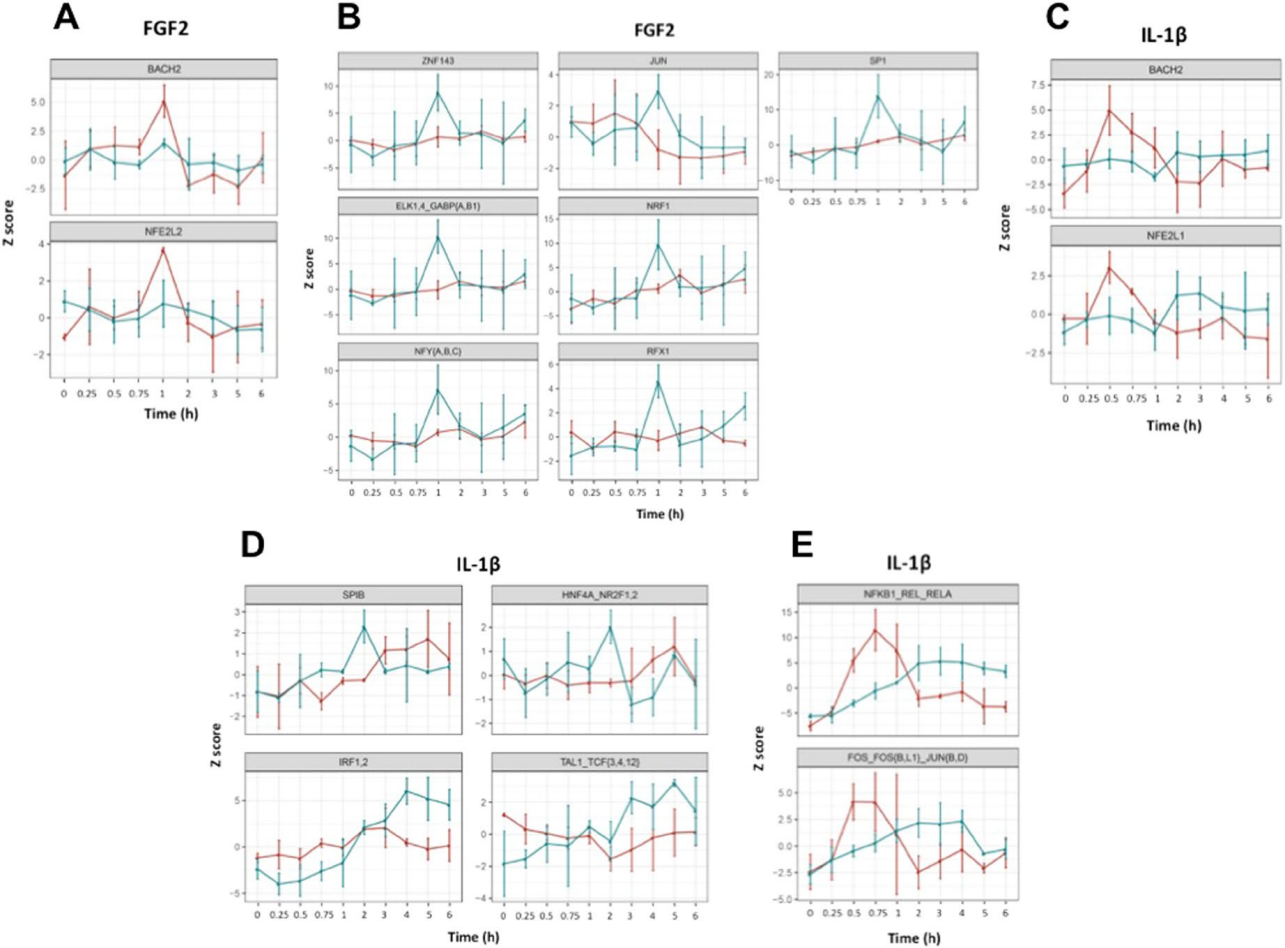
MARA showing dynamic changes in motif activity in response to FGF2 or IL-1β. (**A**) Motifs with dynamic activity in enhancers only in response to FGF2. (**B**) Motifs with dynamic activity in promoters only in response to FGF2. (**C**) Motifs with dynamic activity in enhancers only in response to IL-1β. (**D**) Motifs with dynamic activity in promoters only in response to IL-1β. (**E**) Motifs with activity that peaks in enhancers before promoters in response to IL-1β. Data refer to enhancer (red) and promoter (green).

MARA also enabled categorization of dynamic change in motif activity. For example, in SMC exposed to FGF2 motifs with dynamic activity in enhancers but not promoters include BACH2 and NFE2L2 (Fig. 4A). Motifs with dynamic activity in promoters but not enhancers include ZNF143, NRF1, ELK1,4_GABP{A,B1]}, RFX1, NFY{A,B,C}, SP1 and JUN (Fig. 4B). In SMC exposed to IL-1β, motifs with dynamic activity in enhancers but not promoters include BACH2 and NFE2L1 (Fig. 4C) and motifs with dynamic activity in promoters but not enhancers include SPIB, IRF1,2, HNF4A_NR2F1,2 and TAL1_TCF{3,4,12}^27^ (Fig. 4D). Interestingly, in IL-1β exposed cells motifs with activity that peaks in enhancers before a peak in motif activity in promoters include NFKB1_REL_RELA and FOS_FOS{B,L1}_JUN{B,D} (Fig. 4E) but we could not identify any motifs fitting this category in FGF2 exposed cells. These data imply differences in the SMC response to growth factor or cytokine exposure, and indeed, we have demonstrated the mechanistic importance of ATF4^28,29^ and JUN^30–32^ in SMC pathobiology. These data suggest that certain TFs (such as JUN in response to FGF2 or IRF1,2 in response to IL-1β) are mainly involved in proximal (promoter) regulation of transcription, whereas others (such as NFE2L2 in response to FGF2 or FOS_FOS{B,L1}_JUN{B,D} in response to IL-1β) are mainly involved in distal (enhancer) regulation.

## Discussion

When SMC are exposed to mitogenic or pro-inflammatory factors such as FGF2 or IL-1β, dynamic changes in molecular signaling and transcription ensue. This in turn may lead to autocrine and paracrine growth, and the eventual formation of SMC-rich intimal lesions in the case of restenosis or the evolution of atherosclerotic lesions that can later involve a destabilizing inflammatory component. These events are thought to be triggered by the activity of IEG that are rapidly and transiently stimulated by growth factors or cytokines that fuel the growing lesion. Understanding similarities and differences in patterns of gene expression controlled by growth factor or cytokine exposure provides important comparative insights on specific genes regulated by each agonist in SMC. Here we exploited CAGE analysis to characterize differences in promoter usage and promoter dynamics in primary human SMC exposed to FGF2 or IL-1β. The CAGE technique allows high throughput analysis of gene expression by identifying sequence tags corresponding to 5′ ends of mRNA transcripts at cap sites and TSS, and FANTOM5 served to map many human TSS and associated promoters in a cell- or condition-specific context. Although in the present study FGF2 and IL-1β were used as model agonists, several other pathophysiologically-relevant factors such as platelet-derived growth factor, transforming growth factor or other interleukins could also have been used but were not due to limited resources. For example, PDGF-DD has been compared with IL-1β at a single time point (24 h) in rat SMC by way of gene chip expression analysis^33^. While the concentrations of FGF2 and IL-1β used here have been used by others with SMC^34,35^, notwithstanding possible differences in receptor type or number, it is possible that the promoter kinetics observed with FGF2 and IL-1β may be dose related. In the PDGF-DD versus IL-1β example, 30 ng/ml of PDGF-DD was compared with 2.5 ng/ml of IL-1β. IL-1β distinctly increased 672 genes, PDGF-DD distinctly increased 515 genes and 88 genes were increased by both. Conversely, IL-1β distinctly reduced 527 genes, PDGF-DD distinctly reduced 317 genes and 206 genes were reduced by both^33^.

There were differences in IEG promoter usage and dynamics in SMC exposed to FGF2 or IL-1β. p1@EGR-1 has a rapid short response and a late response to FGF2, whereas p2@EGR-1 has a rapid short response and a late flat response to IL-1β. Similarly, p1@IL8 has a late response or a long response to IL-1β but a late response or an early standard response and a late response or a late flat response to FGF2. p1@FOS has a rapid short response and a late response to IL-1β or FGF2^12^. Cytokines and growth factors trigger signal transduction pathways by way of specific interactions with cell surface receptors. That IL8 is maximally induced by FGF2 at 1 h but maximally induced at 4 h by IL-1β indicates that different molecular mechanisms mediate IL8 induction by the growth factor and cytokine. K-means clustering revealed that in response to SMC exposure to FGF2 or IL-1β, there were sets of genes induced, repressed or unchanged. This identified genes, such as EGR-1, FOS, JUN and KLF5/BTEB2, known mediators of vascular pathobiology. Moreover, there are interdependencies among these TFs in vascular SMC. KLF5/BTEB2 is a target of EGR-1^36^ and JUN controls EGR-1^31,37^.

MARA is a key inferential technique that was used here to link TF binding motifs with dynamic transcriptional responses to FGF2 or IL-1β as performed by a number of other groups including studies of virus infection^38^ or responsiveness to LPS^39^. Cluster analysis after MARA revealed that most TF recognition motifs in SMC exposed to FGF2 were associated with a sharp inducible response at 1 h whereas most motifs in SMC exposed to IL-1β were associated with a biphasic response initially dipping at 15 min and peaking generally later than the FGF2 response between 1–5 h. This biphasic response likely reflects different roles that these motifs play following cytokine exposure. MARA also suggests that certain TFs are principally involved in proximal regulation of transcription, whereas others are mainly involved in distal regulation. MARA is not particularly sensitive for the detection of activity of factors interacting with GC-rich motifs within CpG island promoters. For example, the GC rich EGR-1..3 motif recognised by C_2_H_2_ zinc fingers in Egr-1 showed little if any change in response to FGF2 or IL-1β by MARA, yet p1@Egr-1 (Supplementary Fig. 1A,C) and p2@Egr-1 (Supplementary Fig. 1B,D) are induced by FGF2 and IL-1β peaking within 30–60 min by CAGE analysis (Supplementary Table 1) and Egr-1 mRNA is induced by FGF2 and IL-1β peaking within 30–60 min by qRT-PCR^12^. The insensitivity of MARA with GC-rich motifs was also recognised by ref.^39^ in monocyte-derived macrophage cells.

Dynamic responsiveness to growth factor or cytokine exposure in growth quiescent SMC supports the notion that vascular injury triggers diverse gene regulatory pathways culminating in a dedifferentiated SMC phenotype. SMC are not terminally differentiated cells and can change phenotype depending on local environmental cues. The changes described here may contribute to the dedifferentiation process in which SMC transition from a contractile to a synthetic phenotype. The net response at any given point in time will depend on the balance of growth factors, cytokines and other stimuli in the local cellular microenvironment. The characterization of IEG into 9 groups depending on their expression profile^12^ should facilitate the identification of links between first responder genes and dependent genes. For example, EGR-1 belongs to the earliest category, the rapid short responder. Previous work by our group showed that EGR-1 is inducible and transiently expressed in the injured artery wall^26^. Egr-1 regulates the expression of at least 300 genes including transcriptional regulators, growth factors and extracellular matrix proteins^40^. In the IL-1β versus PDGF-DD analysis, Egr-1 was induced 1.3- and 4.4-fold, respectively, but this was 24 h after treatment^33^. Egr-1, as an IEG is rapidly and transiently induced. CAGE analysis shows that transient induction of p1@Egr-1 (Supplementary Fig. 1A,C) and p2@Egr-1 (Supplementary Fig. 1B,D) is complete within 2 h.

This study had certain limitations. First, while CAGE sequencing enables identification of alternative transcription start sites, CAGE data may be influenced by post-transcriptional regulation such as miRNA or changes in RNA stability/degradation. Second, this study is limited to *in vitro* investigations of human SMC in culture responding to FGF2 or IL-1β combined with extensive bioinformatics analyses and may not directly apply to SMC phenotypic switching in a broad range of pathological states where signaling pathways and regulatory dynamics may be influenced factors such as type of noxious or mechanical stimuli, protease activity, matrix degradation, mitogen/cytokine release kinetics and receptor interactions in the complex microenvironment of intact arteries. Finally, although FGF2 or IL-1β were used as stimuli, numerous other factors or conditions could have been used within the context of atherosclerotic plaques^41^, including a cholesterol loading which facilitates SMC transdifferentiation into CD68 positive, macrophage-like cells^42^. Future studies should interrogate biological correlates of altered transcription, and include strategies that block or overexpress IEG, changes in cell morphology or physiology, test TF occupancy by ChIP/ChIP-Seq or mutagenesis, or use of animals lacking or overexpressing these genes in genomics analysis of injured arteries.

## Materials and Methods

### Cell culture and siRNA experiments

Samples of primary human SMC were those previously described^12^. Briefly, human aortic SMC (pool of 3 donors) were purchased from Cell Applications (CA, USA) and grown in Waymouth’s medium, pH 7.4, supplemented with 1 mM L-glutamine, 10 U/ml penicillin, 10 µg/ml streptomycin and 10% fetal bovine serum at 37 °C with 5% CO_2_. SMC at passages 3–8 were used for subsequent experiments. Cells were seeded into 10 cm plates and at 80–90% confluence, washed with PBS, pH 7.4 and incubated in serum free medium for 24 h. The cells were incubated in medium containing FGF2 (50 ng/ml, Promega) or IL-1β (10 ng/ml, Calbiochem) for 15, 30, 45, 60, 120, 180, 240, 300 or 360 min. Control (0 min) samples represent cells harvested from serum deprived and unstimulated. Total RNA was harvested using Trizol reagent method (Invitrogen, Carlsbad, CA, USA) followed by RNA extraction using RNeasy Mini Kit (Cat# 74104, Qiagen) according to the manufacturer’s protocol. DNase reagent was used to eliminate DNA contamination of the RNA (Invitrogen). RNA was sent to RIKEN Yokohama Institute for CAGE analysis.

Dharmacon ON-TARGET plus SMART pool siRNA (pool of four siRNA, Cat# L-006526-00-0050) specific for human EGR-1^43^ was purchased from Thermo Scientific. Dharmacon ON-TARGET plus Non-Targeting Control Pool (siCTL, Cat# D-001810-10-50) also was purchased from Thermo Scientific and was used in this study to validate siRNA specificity.

SMC were serum starved for 6 h before transfection with siRNA for 20 h. A master mix of 2.68 µl DharmaFECT 2 (Thermo Scientific) for every 1 µg of siRNA was made in Waymouth’s medium containing 0.05% FBS (without antibiotics) and the master mix was incubated at room temperature for at least 20 min to complex the siRNA and liposomes. The Master Mix was then added to the culture in a drop-wise manner, mixed by gentle swirling and placed back into the incubator at 37 °C for 20 h. In total, cells remained under serum arrest conditions for 26 h.

### Western blot analysis

Western blot analysis was performed with extracts of cells treated with siRNA targeting human EGR-1. SMC (80–90% confluency) were arrested in Waymouth’s medium (Invitrogen, MD) containing 0.05% FBS for 6 h. Cells were incubated with 100 nM EGR-1 siRNA, siCTL or the transfection agent DharmaFECT alone overnight. FGF2 (50 ng/ml) was added for 1 h. Total protein was harvested in radioimmunoprecipitation (RIPA) lysis buffer with protease inhibitors^44^. Proteins were resolved on 4–20% (w/v) sodium dodecyl sulfate (SDS)-polyacrylamide gradient gels (Bio-rad Mini-PROTEAN TGX) and transferred to Immobilon-P PVDF membranes (Millipore, USA). Membranes were blocked with 5% skim milk and incubated with primary rabbit monoclonal EGR-1 antibody (1:1000, Cell Signaling, USA) at 4 °C overnight or mouse monoclonal ß-actin antibody (1:30000, Sigma-Aldrich) at 22 °C for 1 h then incubated with a secondary goat anti-rabbit (1:1000, DAKO Cytomation, Denmark) or goat anti-mouse (1:1000, DAKO Cytomation, Denmark) antibodies for 1 h. Chemiluminescence was detected using the Western Lightning Chemiluminescence system (PerkinElmer, USA) and ImageQuant™ LAS 4000 biomolecular imager (GE Healthcare Life Sciences, USA). Band intensity was quantitated using the Gel Analysis method in NIH ImageJ and normalized to β-actin.

### SMC dual chamber migration assay

SMC (6 × 10^3^ cells) suspended in Waymouth’s medium supplemented with 20% FBS, 1 mM L-glutamine, 10 U/ml penicillin and 10 µg/ml streptomycin were seeded into the upper chamber of 24-well plates fitted with Millicell cell culture inserts (Cat# PI8P01250). After 48 h, the medium was changed to Waymouth’s supplemented with 5% FBS, 1 mM L-glutamine, 10 U/ml penicillin and 10 µg/ml streptomycin and the cells were incubated for 24 h. siRNA targeting human EGR-1 or siCTL or DharmaFECT only were prepared in Waymouth containing 5% FBS and 1 mM L-glutamine (no antibiotics) and added to the upper chamber and incubated overnight. FGF2 (50 ng/ml, PEPROTECH, Cat# 100-18B) in medium containing 5% FBS was added to the lower chamber. After 48 h, medium from the upper chamber was removed and a cotton swab was used to remove non-migrated cells and excess liquid. The insert was placed in 70% ethanol for 10 min to allow cell fixation and membranes were dried for 10–15 min. Filters were excised, placed onto slides, mounting medium (Fluoroshield™ with DAPI, Sigma, Cat# 6057) was added and specimens were visualized using an EVOS FL microscope. Methods were carried out in accordance with relevant guidelines and regulations, including negligible risk approval by the UNSW Human Research Ethics Committee.

### Cap analysis gene expression (CAGE), response patterns and MARA

Tag-cluster expression, differential expression and response patterns are from ref.^12^. CAGE captures and sequences cDNAs corresponding to the 5′ ends of capped RNAs without relying on 20–21 nt fragments or PCR amplification^13,15,45^. Data that did not meet quality control requirements in the CAGE library (a RNA integrity number (RIN) score of at least 6 was required for inclusion)^12^ were excluded from further analysis. SMC stimulated with FGF2 for 4 h were not included in this study since RIN criteria were not met. A list of samples passing quality control and analysed in this study is provided in Supplementary Table 4. Promoters were defined as the robust set of CAGE peaks identified by the decomposition peak identification (DPI) method in the FANTOM5 project^12,15^. Promoters were associated to gene symbols and TFs as described in ref.^15^. The notation p1@<GENE_NAME> corresponds to the <GENE_NAME> promoter that has the highest tag support across all FANTOM5 samples, whereas p2@<GENE_NAME> signifies the promoter with the second most tag support etc. For each pair of time points, differentially expressed promoters were identified using edgeR^46^ using default parameters and common dispersion, on promoters having at least 5 CAGE tags across the time course, with promoters having a *q*-value (false discovery rate) below 0.05 assigned as differentially expressed. Enhancers were defined as described in refs^12,47^; briefly, enhancers are identified as bidirectionally transcribed loci having CAGE expression on both strands across all FANTOM5 samples at least 500 bp from annotated genes. The resulting data is accessible at the FANTOM5 web resource^48^ and ZENBU (http://fantom.gsc.riken.jp/zenbu/) was used for manual inspection^49^. Log2FC was calculated by obtaining average expression of a time point then calculating the log2FC vs t0 using a pseudocount of 1 TPM. By calculating log2 fold changes we assumed that up and down regulation would be symmetric (meaning the equivalent down regulation to a up regulation of log2FC = 2 is log2FC = −2). Motif activity response analysis (MARA) was performed as described^12^. Briefly, TFs are assumed to regulate the expression of promoters through binding to DNA sequence elements in proximal regions (−300 bp to +100 bp from the representative CAGE peak in the promoter). The expression of a promoter in a sample is assumed to be a linear function of the number of conserved TF binding sites in the proximity of the promoter. Specifically, we assume that

$${e}_{p,s}=noise+{c}_{p}+{c}_{s}+\,\sum _{m}({N}_{p,m}\ast {A}_{m,s})$$

where *e*_*p,s*_ is the logarithm of the expression of each promoter *p* in sample *s*, the noise is assumed to be normally distributed with the same standard deviation for all promoters in the sample, *c*_*p*_ is a promoter dependent constant, *c*_*s*_ is a sample dependent constant, and *N*_*p,m*_ is the predicted number of functional binding sites for motif *m* that appear in promoter *p*. The expression level was determined by CAGE, and the motif activities of known motifs (SwissRegulon^50^) are fitted to the data using all promoters that are expressed with at least ten tags per million (TPM) in at least one of the samples. The motif activities represent sample-dependent abilities of motifs to regulate expression levels. Five clusters capturing patterns of motif activities and 10 clusters of promoter activities in k-means clustering were chosen based on patterns across the different FANTOM5 time courses^15^.

### Statistical analysis

Statistical analysis was performed using one-way ANOVA where a P value of <0.05 was considered significant.

## Electronic supplementary material

Supplementary Information

Suppl Table 1-Responsive genes

Suppl Table 2-Transcription factor clusters FGF2 & IL1

Suppl Table 3A-MARA clusters FGF2

Suppl Table 3B-MARA clusters IL1

Suppl Table 4-SMC_sample_list

Supplementary Information

## Data Availability

The datasets generated and/or analysed during the current study are available in the FANTOM5 data repository (http://fantom.gsc.riken.jp/5/data/), as Supplementary Information files or from the corresponding author on reasonable request.
