# Supplementary material for: Promoter Usage and Dynamics in Vascular Smooth Muscle Cells Exposed to Fibroblast Growth Factor-2 or Interleukin-1β
Source: Sci Rep. 2018 Sep 3;8:13164. doi: 10.1038/s41598-018-30702-4 (PMC6120868; doi:10.1038/s41598-018-30702-4)

Supplementary Information

Promoter Usage and Dynamics in Vascular Smooth Muscle Cells Exposed to Fibroblast Growth Factor-2 or Interleukin-1β

Ahmad M. N. Alhendi, Margaret Patrikakis, Carsten O. Daub, Hideya Kawaji, Masayoshi Itoh, Michiel de Hoon, Piero Carninci, Yoshihide Hayashizaki, Erik Arner, Levon M. Khachigian

Supplementary Figure Legend

Supplementary Figure 1. p1@EGR-1 and p2@EGR-1 CAGE in SMC exposed to FGF2 or IL-1β. Growth quiescent SMC were exposed to FGF2 (50 ng/ml) or IL-1β (10 ng/ml) for various times up to 6 h. Fold change CAGE data for p1@EGR-1 (**A, C**) and p2@EGR-1 (**B, D**) sourced from Ref [12]. Samples at 0 min represent serum starved, unstimulated samples. Significance was determined using edgeR software in Ref [12]. Error bars represent SEM.

Supplementary Table Legends

Supplementary Table 1. Promoters regulated in SMC by FGF2 and/or IL-1β. These promoters are differentially expressed significantly (5% FDR threshold by edgeR software) in at least one of all pairwise comparisons of all time points up to and including 6 h.

Supplementary Table 2. Dynamic clustering of TF expression in SMC exposed to FGF2 or IL-1β.

Supplementary Table 3. Motifs associated with dynamic clustering in SMC exposed to (A) FGF2 or (B) IL-1β.

Supplementary Table 4. Samples passing quality control analysed in this study.

Supplementary Figure 1

Promoter Usage and Dynamics in Vascular Smooth Muscle Cells Exposed to Fibroblast Growth Factor-2 or Interleukin-1β

Ahmad M. N. Alhendi, Margaret Patrikakis, Carsten O. Daub, Hideya Kawaji, Masayoshi Itoh, Michiel de Hoon, Piero Carninci, Yoshihide Hayashizaki, Erik Arner, Levon M. Khachigian

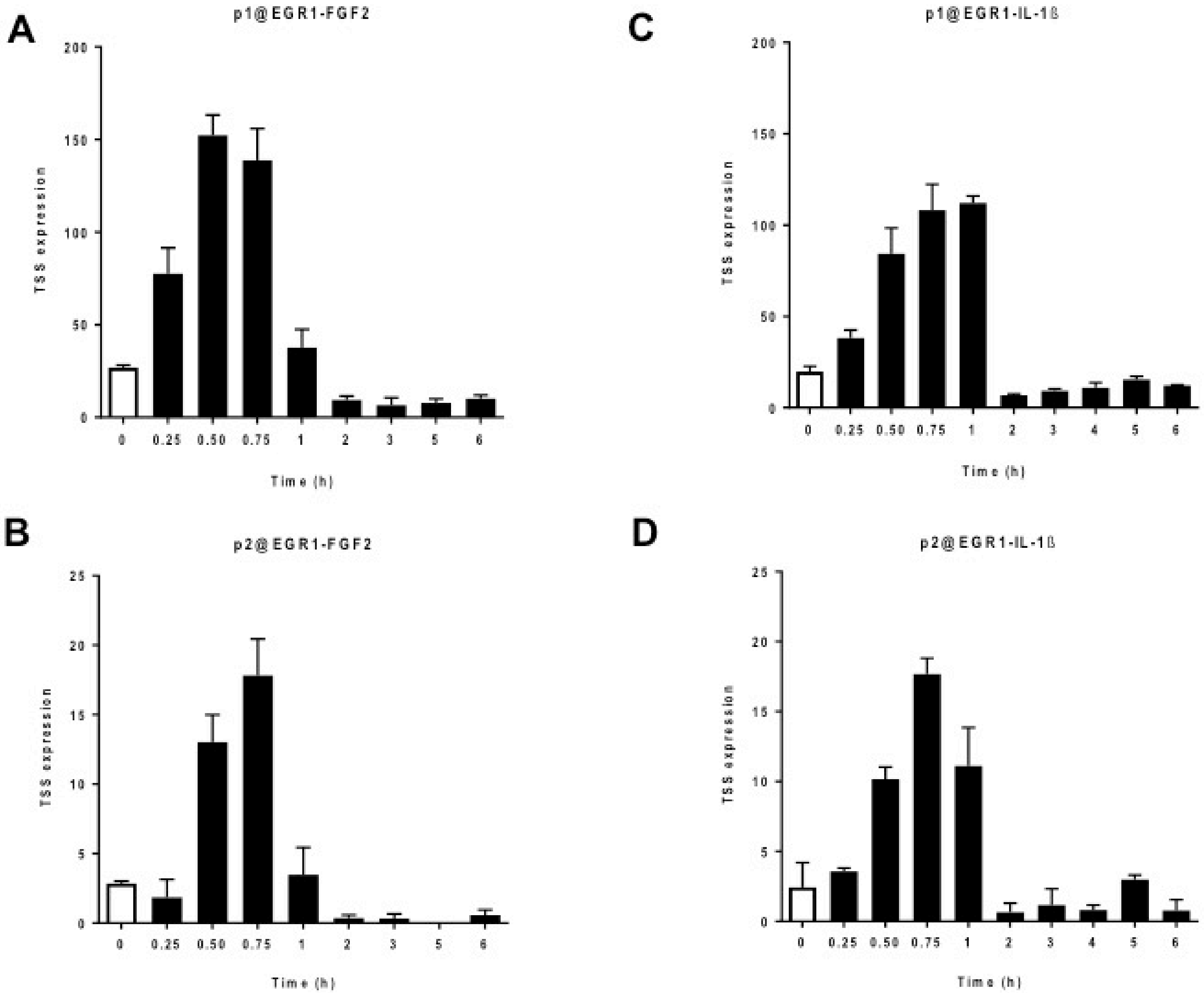

Supplement: Supplementary file 1 — Supplementary Information [file 41598_2018_30702_MOESM1_ESM.pdf]
